# The potential for coproduction to add value to research

**DOI:** 10.1111/hex.12821

**Published:** 2018-08-31

**Authors:** Dr. Gary Hickey

**Affiliations:** ^1^ National Institute for Health Research INVOLVE Southampton United Kingdom

Coproduction has a rich history and has been applied and developed in a range of disciplines.^1^ The term has been used for several decades^1^ and is usually associated with the design and improvement of services.^2^, ^3^ It offers the potential to evolve and improve public involvement in research—a means of further ensuring that the public are active collaborators in research^4^. Much of the drive comes from a perceived opportunity for coproduction to more closely align research and innovation with the values, needs and expectations of society.^5^

Given the various ways in which coproduction has been interpreted and applied, it is hardly surprising that it is a contested concept with much confusion about what it is and how one “does” it.^6^ For example, for some coproduction is simply “good” public involvement in research, for some it is a vogue term that has been applied loosely to existing approaches to public involvement in research, and for still others it is a particular methodology.^7^ Despite this lack of clarity, research is being coproduced with the public and just as public involvement in research generally is now an international phenomena^8^ so is the coproduction of research.

The National Institute for Health Research (funded by the Department of Health and Social Care to improve the health and wealth of the nation through research) has committed to exploring how coproduced research might work in practice in health and social care research in England.^9^ To this end, INVOLVE has led on the development of guidance which is intended to provide greater clarity about what it means to coproduce research.^10^ There is no one set way of coproducing research. Rather, it is principle‐driven and can take a variety of formats including partnerships between academia and organizations representing the public as well as members of the public being employed by organizations which undertake research, for example universities. The key principle involved in coproducing research is the sharing of power in key decisions. No longer do researchers and/or practitioners only own the decisions and the research. Relationships need to be valued and developed and maintained. Efforts need to be made to address power differentials.

*“*Co‐producing a research project is an approach in which researchers, practitioners and the public work together, sharing power and responsibility from the start to the end of the project, including the generation of knowledge*.”* 10

On the one hand, coproduction can be viewed as another approach to public involvement which sits alongside other approaches such as consultation, collaboration and user‐controlled research.^11^ In practice of course there are often blurred boundaries between these approaches—and research can be a dance moving back and forth between approaches. For example, consultative approaches can merge into collaboration, and vice versa. Any framework then that seeks to distinguish between approaches is best seen as an analytical tool through which we can view and understand our world. However, coproduction is also an approach to research that goes beyond public involvement—it has principles that apply across the team and underpin the way the research is undertaken.

Guidance on principles and key features are useful in helping us move towards clarity but they do not show us “how” to coproduce or the various challenges that coproducing research presents. How, for example, do we share power when it is often a principal investigator who is accountable for decisions? How do we build the relationships in a research team that ensure that power differentials are addressed? Some suggest that coproduction represents a paradigm shift in research^12^ changing how we determine what to research, how it is undertaken, and how knowledge is generated. It challenges power structures and the way in which research is currently funded and governed; power is shared across those involved in the research; and plans are more likely to be emergent. It challenges what we mean by impacts—in coproduced research as much emphasis is placed on impacts that emerge from the process of undertaking the research, for example expanded social networks, as there is on the impacts of the outcomes of the research. It challenges what we mean by knowledge and research—the collection and analysis of empirical data is just one form of the generation of knowledge.

This edition of Health Expectations is timely, providing some examples of patient/consumer involvement and engagement in research.

The Editorial team also wish to solicit papers for a special issue of Health Expectations on Patient and Public Involvement and Engagement (PPIE) in health service provision and research. These paper submissions may include high‐quality systematic review and original research papers reporting aspects of PPIE and coproduction with a particular focus on developing and emerging economies. If your research meets the above criteria, please consider submitting your work to Health Expectations. For further information, please see https://onlinelibrary.wiley.com/journal/13697625.
